# Assessing the reliability of uptake and elimination kinetics modelling approaches for estimating bioconcentration factors in the freshwater invertebrate, *Gammarus pulex*

**DOI:** 10.1016/j.scitotenv.2015.12.145

**Published:** 2016-03-15

**Authors:** Thomas H. Miller, Gillian L. McEneff, Lucy C. Stott, Stewart F. Owen, Nicolas R. Bury, Leon P. Barron

**Affiliations:** a Analytical & Environmental Sciences Division, Faculty of Life Sciences and Medicine, King's College London, 150 Stamford Street, London SE1 9NH, United Kingdom; b AstraZeneca, Global Environment, Alderley Park, Macclesfield, Cheshire SK10 4TF, United Kingdom; c Division of Diabetes and Nutritional Sciences, Faculty of Life Sciences and Medicine, King's College London, Franklin Wilkins Building, 150 Stamford Street, London SE1 9NH, United Kingdom

**Keywords:** Pharmaceuticals, Pesticides, Toxicokinetics, Bioconcentration, Invertebrates

## Abstract

This study considers whether the current standard toxicokinetic methods are an accurate and applicable assessment of xenobiotic exposure in an aquatic freshwater invertebrate. An *in vivo* exposure examined the uptake and elimination kinetics for eight pharmaceutical compounds in the amphipod crustacean, *Gammarus pulex* by measuring their concentrations in both biological material and in the exposure medium over a 96 h period. Selected pharmaceuticals included two anti-inflammatories (diclofenac and ibuprofen), two beta-blockers (propranolol and metoprolol), an anti-depressant (imipramine), an anti-histamine (ranitidine) and two beta-agonists (formoterol and terbutaline). Kinetic bioconcentration factors (BCFs) for the selected pharmaceuticals were derived from a first-order one-compartment model using either the simultaneous or sequential modelling methods. Using the simultaneous method for parameter estimation, BCF values ranged from 12 to 212. In contrast, the sequential method for parameter estimation resulted in bioconcentration factors ranging from 19 to 4533. Observed toxicokinetic plots showed statistically significant lack-of-fits and further interrogation of the models revealed a decreasing trend in the uptake rate constant over time for rantidine, diclofenac, imipramine, metoprolol, formoterol and terbutaline. Previous published toxicokinetic data for 14 organic micro-pollutants were also assessed and similar trends were identified to those observed in this study. The decreasing trend of the uptake rate constant over time highlights the need to interpret modelled data more comprehensively to ensure uncertainties associated with uptake and elimination parameters for determining bioconcentration factors are minimised.

## Highlights

• Toxicokinetics for eight pharmaceuticals are presented in *Gammarus pulex*.
• Bioconcentration factors ranged from 12 to 4533 and depended on the method used.
• Decreasing trends in the uptake (*k*_1_) rate constants were observed.
• Recognised models and their assumptions may lead to BCF estimation inaccuracies.
• Meta-analysis of previous toxicokinetic work revealed similar trends in *k*_1_.

## Graphical abstract

**Figure:**
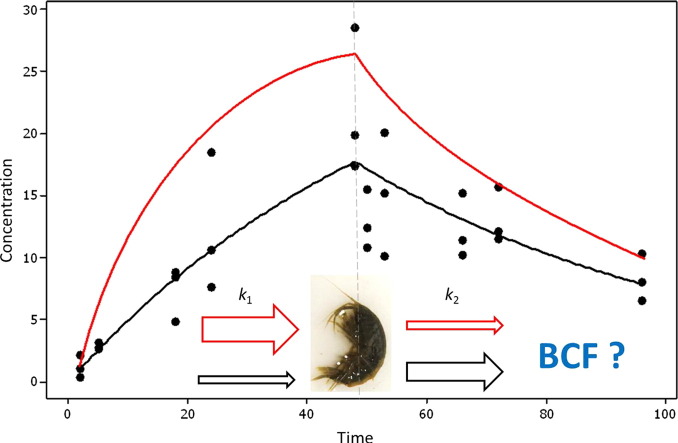

## 1 Introduction

The pseudo-persistent nature of pharmaceuticals and personal care products (PPCPs) has been highlighted in recent years as an environmental concern and has led to the introduction of a watch list under the EU Water Framework Directive which includes an anti-inflammatory, diclofenac, and two hormones, the synthetic ethinyl-estradiol (EE2) and natural estradiol (2013/39/EU, 2013). Several thousand PPCPs are currently available worldwide and whilst measured environmental concentrations typically range from low ng L^− 1^ to high μg L^− 1^, their potential to effect an ecotoxicological response and/or bioaccumulate in a range of biota still remains understudied (De Lange et al., 2006, Contardo-Jara et al., 2011).

Ecotoxicological studies have shown that measured PPCP concentrations in surface waters would be highly unlikely to cause acute effects on exposed organisms (Crane et al., 2006). However, chronic exposure has been linked to behavioural activity changes, increased oxidative stress and alterations to the function of several vital organs in fish and invertebrates (Heckmann et al., 2007, Fernández et al., 2013). Aquatic invertebrates such as molluscs and smaller crustacean species have been previously utilised for monitoring PPCPs in the natural aquatic environment. Most recently, the freshwater amphipod, *Gammarus pulex*, was found to contain residues of carbamazepine, diazepam, nimesulide, trimethoprim and warfarin measuring at low ng g^− 1^ concentrations in UK streams (Miller et al., 2015). PPCP uptake has also been previously observed at low ng g^− 1^ concentrations in wild and caged mussel species collected from the coast of Ireland, the Bohai Sea in China, the Mediterranean Sea and San Francisco Bay, highlighting the extent of PPCP contamination worldwide (McEneff et al., 2014, Li et al., 2012, Bueno et al., 2013, Klosterhaus et al., 2013). EU Directive 93/39/EEC requires an environmental risk assessment to be carried out prior to drug licencing in order to determine any significant toxicological risks associated with a xenobiotic (Straub, 2002). Under the regulatory guidelines, environmental toxicity testing of pharmaceuticals requires standard acute toxicity tests, such as LC_50_ testing, to be carried out unless the predicted environmental concentration (PEC)/predicted no effect concentration (PNEC) ratio is < 1, whereby no further toxicity testing is required. Standardised toxicity tests on aquatic organisms are generally limited to algae (*Desmodesmus subspicatus* or *Pseudokirchneriella subcapitata*), *Daphnia magna* and/or fish (e.g. *Danio rerio*) considered as good model species from freshwater environments. Furthermore, a lack of published research generally exists on the uptake and depuration kinetics of PPCPs both in target and non-target aquatic species to help elucidate potential acute versus chronic effects.

Toxicokinetic studies identify whether a compound will accumulate to potentially toxic levels in/on the organism itself over time or potentially act as a source of toxicity in higher trophic organisms (Ashauer & Escher, 2010). In aquatic species, this can involve the study of either accumulation of compounds via water exposure only (i.e. bioconcentration), or via exposure through both water and diet (i.e. bioaccumulation or biomagnification) (Oliver and Niimi, 1983, Meador et al., 1995). Fish exposure studies often allow a time period for the compound of interest to reach steady-state within the organism, where the rate of uptake is equal to the rate of depuration. However, this time can vary considerably and has led to the application of kinetic modelling where uptake and elimination rates are estimated and used to derive a bioconcentration factor (BCF) (Veith et al., 1979). This factor can be determined in two ways: (a) as a ratio of either the compound concentrations in the organism and the water phase at steady-state, or (b) as the ratio of the uptake (*k*_1_) and elimination (*k*_2_) rate constants (Kenaga, 1972). This approach has been widely evaluated in the literature. Earlier models, such as those for methylmercury in fish (Norstrom et al., 1976) considered several variables including volume of water passing the gills, assimilation across the gills and body weight of the organism. More recent models have been developed to also account for water-phase and lipid-phase resistance, fish lipid content and compound log*K*_ow_ (Veith et al., 1979, Gobas and MacKay, 1987, Hendriks and Heikens, 2001). A widely known and accepted model used to calculate the bioaccumulation of a compound in fish via aqueous and dietary exposure is outlined in the Organisation for Economic Co-Operation and Development (OECD) 305 guidelines (OECD, Test No. 305). These guidelines present two methods for estimation of *k*_1_ and *k*_2_. The sequential method can be performed in one of two ways, a *k*_2_ value can be estimated by linear regression and then curve fitting methods are applied to find *k*_1_. Alternatively, curve fitting methods can be used to estimate *k*_2_ first which is then used to estimate *k*_1_. The simultaneous model calculates both *k*_1_ and *k*_2_ together and is considered a potentially more reliable and realistic model for concurrent uptake and elimination processes occurring in biological systems. Considering the number of PPCPs available on the market that may require testing under EU REACH legislation (European Commission, 2006), the time scales (2 week acclimatisation followed by 28 days for the uptake phase alone unless steady-state is achieved sooner) and number of organisms required for each test (n = 4 per time-point for each exposure) the testing regime to apply to all chemicals under REACH would appear unfeasible. Furthermore, current policy aims to reduce the number of fish used for scientific research, thus current methods proposed such as the OECD guidelines should account for this more ethical approach (Carter et al., 2014, Browne, 2013). Several recent studies assessing BCF have utilised shorter exposure times with experiments lasting only 4–7 days using aquatic invertebrates as a means to assess the potential for substance to bioaccumulate in aquatic organisms (Ashauer et al., 2006, Meredith-Williams et al., 2012). These ecotoxicological studies are important to direct future risk assessment and essential when considering contaminant monitoring in water, sediment and biota.

In the present study, an *in vivo* experiment was carried out to determine the uptake and depuration kinetics of environmentally relevant (low μg·L^− 1^) concentrations of several selected PPCPs in the common freshwater invertebrate, *G. pulex*, using radioactive labels and liquid scintillation analysis. Lastly, the OECD 305 guidelines currently used for modelling of uptake and elimination kinetics in aquatic species are critically evaluated for the first time based on the results obtained both in this study and other published works on micropollutants.

## 2 Materials and methods

### 2.1 Reagents, chemicals and consumables

Radio-labelled pharmaceuticals including ^3^H-propranolol hydrochloride (29.0 Ci mmol^− 1^) were acquired from Amersham Biosciences. ^3^H-metoprolol (29.7 Ci mmol^− 1^), ^3^H-formoterol (18.5 Ci mmol^− 1^) and ^3^H-terbutaline (29.0 Ci mmol^− 1^) were obtained from Vitrax. ^14^C-ibuprofen (2.03 Ci mmol^− 1^) was obtained from American Radiolabelled Chemicals Inc. (St Louis, US). ^3^H-ranitidine (2.5 Ci mmol^− 1^) was obtained from Moravek Biochemicals, ^14^C-diclofenac (0.063 Ci mmol^− 1^) and ^3^H-imipramine hydrochloride (48.5 Ci mmol^− 1^) from Perkin-Elmer. All stock solutions were stored in ethanol. Hydrogen peroxide solution (30% w/w) and analytical grade salts (> 99%) including sodium hydrogen carbonate, magnesium sulphate, calcium sulphate, potassium chloride were purchased from Sigma (Dorset, UK). Tissue solubiliser (Solvable™) and liquid scintillation cocktail (Hionic Fluor™) were purchased from Fischer Scientific Ltd. (Loughborough, UK). Ultra-pure water was obtained from a Millipore Milli-Q water purification system with a specific resistance of 18.2 MΩ·cm or greater (Millipore, Bedford, MA, USA). 6-Well culture plates were obtained from VWR (Leicestershire, UK).

### 2.2 Sample collection and culture maintenance

*G. pulex* were collected by kick-sampling from the River Cray, South-East London, UK, 51°23′09.5″N 0°06′32.4″E. This site was previously shown to have low pharmaceutical contamination in both collected surface water and animal samples (Miller et al., 2015). The populations were transported to the laboratory in 500 mL Nalgene™ flasks filled with surface water from the sample collection site. Populations were rinsed with artificial freshwater (AFW) and then acclimatised to laboratory conditions (as specified below) for a minimum of 7 days before any exposure experiments were performed. AFW was prepared from 1.15 mM of NaHCO_3_, 0.50 mM MgSO_4_, 0.44 mM CaSO_4_ and 0.05 mM of KCl dissolved in 20 L of ultra-pure water. This water was subsequently aerated for several hours to remove dissolved carbonic acid and maximise the dissolved oxygen concentrations. Each culture tank (n = 8) was filled with 2.5 L of AFW and animals were fed with alder leaves that were previously collected from the sampling site and conditioned by submersion in surface water for two days prior to use.

### 2.3 Toxicokinetic exposure and conditions

Toxicokinetic experiments were performed separately for each pharmaceutical for a total of 96 h which included a 48 h uptake phase followed by a 48 h depuration period. Individual adult organisms, both male and female and each > 5 mg wet weight, were placed in each well of 6-well culture plates. *G. pulex* were carefully transferred to well plates using blunt forceps to avoid any harm to the organisms before exposure. A single well contained one organism in 10 mL of exposure media (AFW and test compound) and only non-parasitised individuals were used (absence of *Pomphorhynchus laevis* indicated by the lack of an orange dot on the dorsal side of the animal). *G. pulex* were exposed to individual PPCPs at a concentration of 1 μg·L^− 1^, except for diclofenac and ibuprofen which were present at 10 μg·L^− 1^. The higher exposure of these two compounds was due to the low activity of the radiolabel. All exposure media contained < 0.05% of solvent (ethanol). A total of 33 organisms were used per exposure and were sampled (n = 3/time-point) at 2, 5, 18, 24 and 48 h in the uptake phase followed by the same time-points in the depuration phase. Along with *G. pulex*, 50 μL water was also sampled from each well for analysis of radioactivity. Each sampled organism was washed in 10 mL of ultra-pure water for 10 s (n = 6) and gently blotted dry to remove any excess exposure media and unbound compound to the cuticle of the animal. Organisms were weighed after sampling to determine body mass and then transferred to scintillation tubes for tissue solubilisation. Three individual organisms were also exposed to unspiked AFW in culture plates and sampled after 96 h in a control experiment to account for any background radiation. Additionally, for each experiment, three wells without *G. pulex* were filled with exposure media to account for losses of the compound by sorption to the walls of culture plates. Culture plates were stored in sealed plastic containers with wet tissue to prevent evaporative losses during the static exposure. The light cycle followed 12:12 h light:dark without a dusk/dawn transition period. All experiments were performed in a temperature controlled room at 15 °C (± 2 °C) and water pH was also measured across each experiment at 8.2 ± 0.1.

### 2.4 Sample preparation and liquid scintillation counting

Water samples (50 μL) collected from each exposure well were added to 2 mL of Hionic Fluor liquid scintillation cocktail and counted for radioactivity on a Beckman LS6500 instrument (Beckman Coulter, Inc.). Sampled *G. pulex* individuals were placed in a scintillation tube with 2 mL of tissue solubiliser and maintained at room temperature (approx. 20 °C) for 96 h. Samples were shaken vigorously and then a 50 μL aliquot of the solubilised biotic extract was added to 2 mL of Hionic Fluor to be counted. To account for any difference in counts caused by colour quenching, hydrogen peroxide (200 μL) was added to a previously counted biotic extract and re-analysed. No difference in counts was observed with or without the presence of hydrogen peroxide, therefore, all other biotic samples were counted without the addition of hydrogen peroxide. In addition, chemiluminescence accounted for < 0.01% of the overall counts, and was therefore ignored.

### 2.5 Modelling bioconcentration factors

Parameter estimation of uptake rate constant (*k*_1_) and depuration rate constant (*k*_2_) was performed using a curve fitting algorithm via Minitab statistical software (Minitab Ltd., Coventry, UK) and as outlined in the OECD 305 Fish Bioconcentration Guidelines (OECD, Test No. 305). The concentration of compound in the organism is assumed to follow first order kinetics and is expressed in Eq. (1),

(1) $$\frac{dC_{\text{organism}}}{dt}=k_{1}\times \left[C_{\text{water}}\right]-k_{2}\times \left[C_{\text{organism}}\right]$$

where, d*C*_organism_/d*t* is the rate of change in the concentration of a compound within/on *G. pulex* (mg kg^− 1^ day^− 1^), *k*_1_ is the uptake rate constant (L kg^− 1^ day^− 1^), *k*_2_ is the elimination rate constant (day^− 1^), *C*_water_ is the concentration in the water (mg L^− 1^) and *C*_organism_ is the concentration in the organism (mg kg^− 1^). Eq. (1) was integrated into Eqs. (2), (3) for fitting of curves to the uptake and depuration data. This method, known as the Levenberg–Marquardt algorithm, uses an iterative formula to minimise the residual errors between the observed and predicted data points and simultaneously estimates *k*_1_ and *k*_2_ values from the fitted curve i.e.

(2) $$\left[C_{\text{organism}}\right]=\left[C_{\text{water}}\right]\times \frac{k_{1}}{k_{2}}\times \left(1-e^{-k_{2}t}\right),\;\text{when}\;0<t<t_{e}$$

(3) $$\left[C_{\text{organism}}\right]=\left[C_{\text{water}}\right]\times \frac{k_{1}}{k_{2}}\times \left(1-e^{-k_{2}\left(t-t_{e}\right)}-e^{-k_{2}t}\right),\;\text{when}\;t>t_{e}$$

where, *t* is the time (days) and *t*_*e*_ is the end time of the uptake phase (days). At steady-state, the rate of uptake should be equal to the rate of depuration and there should be no overall change in analyte concentration within *G. pulex*, as expressed by Eq. (4),

(4) $$k_{1}\times \left[C_{\text{water}}\right]=k_{2}\times \left[C_{\text{organism}}\right]↔\frac{k_{1}}{k_{2}}=\frac{\left[C_{\text{fish}}\right]}{\left[C_{\text{water}}\right]}=\mathrm{BCF}\;$$

where, BCF is the bioconcentration factor (L kg^− 1^). BCF can also be estimated using a sequential method where a simple linear regression model is developed based on the depuration data only. With the assumption of first order kinetics, the model should fit a straight line and its slope represents the elimination rate constant as shown in Eq. (5), i.e.

(5) $$\ln\left[C_{\text{organism}}\right]=-k_{2}\times t+c$$

where, ln[*C*_organism_] is the natural log of the analyte concentration within *G. pulex* and *c* is the intercept, which here equals the natural log of the analyte concentration in the *G. pulex* at the start of the depuration phase. The *k*_2_ from Eq. (5) can then be used as a parameter in the curve fitting algorithm to estimate *k*_1_. The rearrangement of Eq. (2) allows the value for *k*_1_ to be calculated over the time interval specified, as shown in Eq. (6) (Crookes & Brooke, 2011). The assumptions of the equation are that analyte concentration in the water and *k*_2_ remain constant. The *k*_2_ used in Eq. (6) was directly estimated by using linear regression of the depuration data to obtain the slope (*k*_2_). The value of *k*_1_ should remain constant over the entire experiment.

(6) $$k_{1}=\frac{\left[C_{\text{organism}}\right]\times k_{2}}{\left[C_{\text{water}}\right]\times \left(1-e^{-k_{2}\times t}\right)}\;$$

For this study, initial parameters for *k*_1_ and *k*_2_ were arbitrarily set at 0.1 in the software with *C*_water_ set in μg L^− 1^, *t* set at 48 h and the maximum number of iterations was set at 200 upon which optimised *k*_1_ and *k*_2_ values were subsequently derived. Confidence intervals (95%) were plotted for curves and the overall model fits were assessed. The lack-of-fit test was calculated in the Minitab software and was used to assess the fit of the line by comparing the variation in response of the replicate data. Lack-of-fit was assessed at a significance level of 0.05. Correlation coefficients (*r*^2^) were evaluated when the sequential method was used to estimate *k*_2_. The distribution coefficient (log*D*) was generated using ACD Labs Percepta software for the interpretation of estimated BCF values. All compound information is displayed in Table S2 of the SI.

## 3 Results and discussion

### 3.1 Uptake and elimination kinetics for selected PPCPs within *G. pulex*

**Fig. 1:**
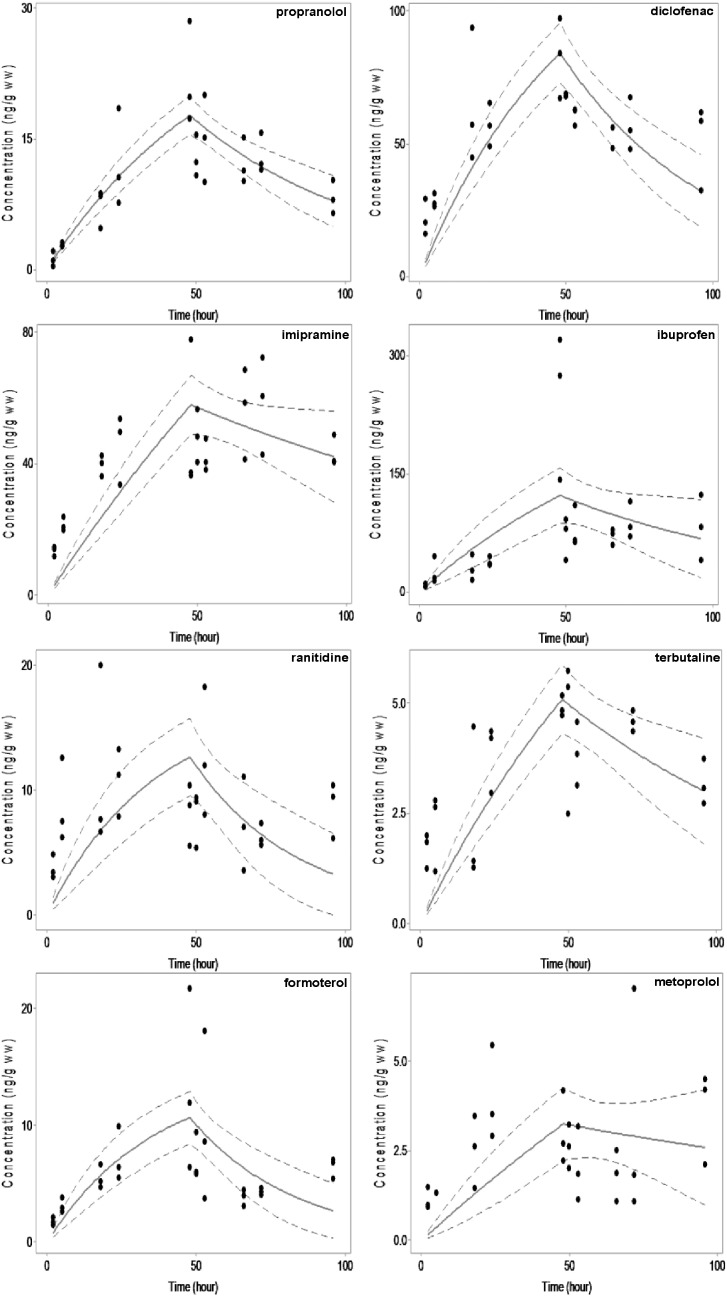
Uptake and elimination data for PPCPs in *G. pulex*. Dashed lines indicate 95% confidence limits.

**Fig. 2:**
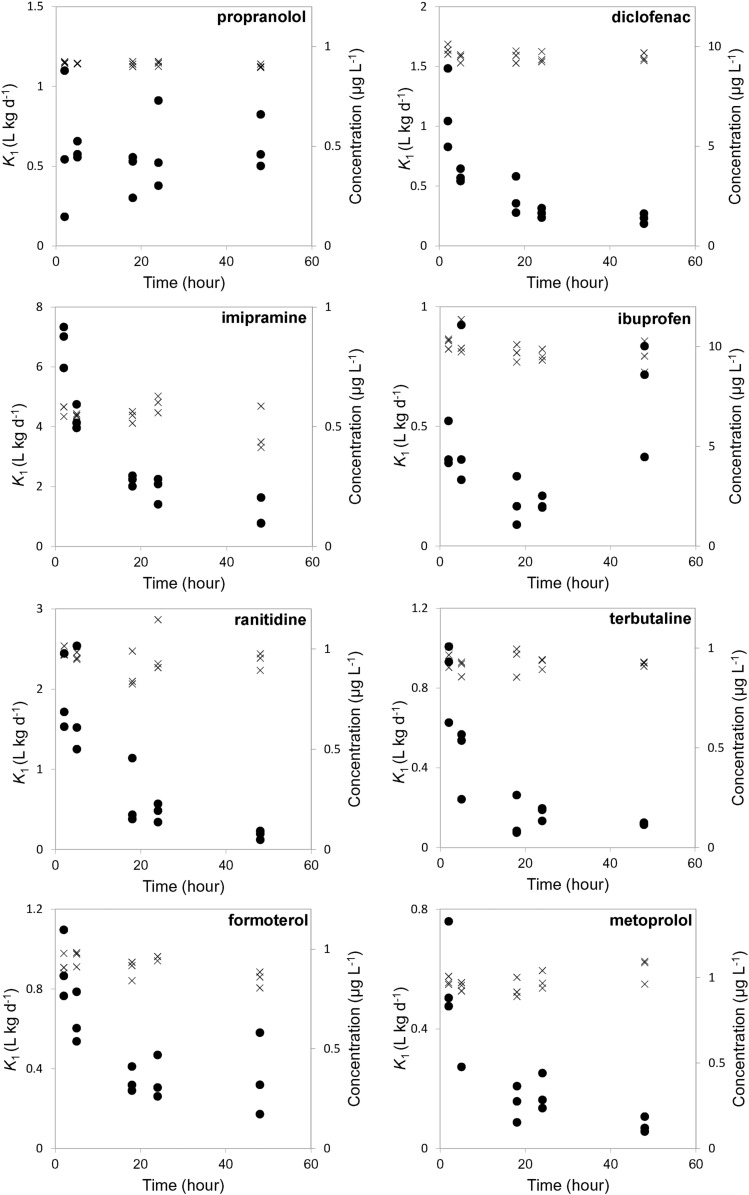
Relationship of uptake rate constants (*k*_1_) over time for eight PPCPs (black circles) and the respective water concentrations (*C*_w_) over time (crosses).

The exposure concentration of each PPCP was selected to approximate the higher ranges of trace pharmaceutical occurrence in the aquatic environment to maintain practically quantifiable limits for reliable analysis (Miller et al., 2015, Hilton and Thomas, 2003, Thomas and Hilton, 2004). Considering that natural uptake and depuration are not separate processes, the BCF values for the selected compounds were determined using the simultaneous model described above (Table 1). Uptake of each pharmaceutical was observed in *G. pulex* as early as 2 h from the point of exposure. The highest residue concentrations measured in *G. pulex* at the 48 h timepoint were ibuprofen and diclofenac, potentially corresponding to the elevated exposure concentrations of 10 μg L^− 1^. All other compounds exposed at 1 μg L^− 1^ measured < 80 ng g^− 1^ ww after 48 h uptake (Fig. 1). The rate of PPCP uptake measured in the exposed *G. pulex* corresponds to the decreases in PPCP concentration measured in the spiked AFW. The largest decrease in PPCP concentration was observed for imipramine, where analyte concentrations in the water decreased to an average of 0.478 μg L^− 1^ corresponding to a 52.2% loss. After 48 h, formoterol concentration also decreased in water by 15% to an average of 0.85 μg L^− 1^. The exposure concentrations of the remaining compounds did not decrease by ≥ 10% (Fig. 2 and Table S1). Additional sources of potential PPCP loss in the aqueous phase should be mentioned and include photolysis, volatilisation, metabolism by microorganisms and sorption to the walls of the exposure well. Of these processes sorption was accounted for by control wells with exposure media only and was shown to account for negligible losses in water concentration except in the case of imipramine (Table S1). Within 2 h, there was a 27% loss of imipramine and within 48 h the loss increased to 39%. As quantification was performed by LSC, any degradation products resulting from transformation or photolysis would contribute towards the total radioactivity and counted as the precursor compound. However, it should be considered that these formed products may potentially have different accumulation potentials and hence latent uptake and elimination kinetics.

Following removal from the contaminated source, relatively high elimination rates were measured for most of the selected compounds. However, imipramine showed increased uptake (*k*_1_ = 1.408 L kg^− 1^ day^− 1^), but lower elimination (*k*_2_ = 0.007 day^− 1^), resulting in the highest BCF value measured at 212. Diclofenac has the same log*P* value as imipramine at 4.4 (log*D*_8.2_ = − 1.1) but attained a significantly lower BCF value of 14 due to its high rate of elimination. Ibuprofen, another acidic drug with a log*P* of 3.5 (calculated log*D*_8.2_ = − 0.1), also had a low BCF value determined at 27. The BCF values for the four compounds with log*P* < 2. (i.e. metoprolol, ranitidine, terbutaline and formoterol) were determined between 12 and 17. Hydrophobicity is generally considered a major factor when determining the bioaccumulation potential of a compound. However, uptake studies related to pharmaceuticals in several species of plants, for example, showed poor correlations between log*D*_ow_ and logBCF and especially so for ionised molecules (Wu et al., 2013). Low bioconcentration of the selected PPCPs was in agreement with a study by Meredith-Williams et al., in which toxicokinetic data for six pharmaceuticals within *G. pulex* was shown with the exception of fluoxetine, a selective serotonin reuptake inhibitor (BCF = 185,900) (Meredith-Williams et al., 2012). In the cases of diclofenac, ibuprofen, imipramine and propranolol, log*P* is similar (3.3–4.7). Therefore, using an uptake model based on hydrophobicity, it would be logical to assume similar uptake rates. A potential reason for their difference could be physicochemical in nature, e.g. due to their anionic or cationic nature as well as the degree of ionisation and log*D*_ow_ value (Erickson et al., 2006). It could also be due to biological factors such as gill surface charge or the boundary layer between the bulk water and the gill surface (Tao et al., 2001). Uptake across the gill may also occur by more than simple passive diffusion for these ionic compounds and thus carrier mediated transport may also have influence on the different ionic species (Sugano et al., 2010, Kell and Oliver, 2014). The increased uptake constants of imipramine, propranolol and formoterol are in agreement with reported gill cell permeabilities to these compounds in the same order of imipramine > propranolol > formoterol (Stott et al., 2015). The low concentrations of PPCP residues measured in the *G. pulex* and unspiked AFW post-exposure highlights the ability for *G. pulex* to readily metabolise and eliminate xenobiotics, as previously shown by Nyman et al. (2014) and Ashauer et al. (2012). This evidence suggests there is conservation of cytochrome P450 enzymes, similarly observed in other aquatic invertebrate species (Solé & Livingstone, 2005).

### 3.2 Comparison of simultaneous versus sequential uptake and depuration process models

Methods used for the calculation of BCF values in *G. pulex* are summarised in Table 1 and also include uptake and elimination constants (± standard error). Many of the toxicokinetic plots in Fig. 1 are shown to have some lack-of-fit. The *p*-value generated from a lack-of-fit test shows that in the simultaneous method there are 5 models that have a statistically significant lack-of-fit indicating potentially inaccurate and unreliable BCF values. It is possible that several large outliers could influence the lack-of-fit test, thus resulting in a statistical significance when potentially none exists. When using the simultaneous method, if a poor fit exists, then the sequential method should be investigated as a potential alternative. The linear regression of the depuration phase data points gives a direct estimate of *k*_2_. The goodness-of-fit is interpreted by visual inspection of the linearity and the *r*^2^ (Fig. S1). Consideration of the sequential method showed an over-estimation of BCF values when compared to the simultaneous model. Deviations from linearity can indicate higher order kinetics. Simple plots of 1/[*C*_organism_] here did not indicate second order kinetics and therefore *k*_2_ values from plots of ln[*C*_organism_] were accepted. Low *r*^2^ values for some compounds were likely due to the scatter in measured internal concentrations. Comparison of derived *k*_2_ values showed that imipramine, formoterol, ranitidine, diclofenac and terbutaline had significantly lower elimination constants in comparison to the simultaneous model approach (Table 1). Markedly reduced estimations in *k*_2_ for imipramine and ranitidine corresponded to a large increase in BCF for ranitidine, increasing 4-fold, and imipramine, increasing > 10-fold to ~4200 on average between curve fitting and linear regression approaches. Given the inherent non-standard method we have applied, further work would be necessary to better understand this apparently high BCF and we would caution reliance on this value from such a limited study. When using a curve fitting method to calculate an elimination constant in the sequential method there was good agreement between the linear regression estimates of *k*_2_, indicating the estimate of *k*_2_ was correct. The p-values for the curve fits indicated that there was only one statistical lack-of-fit for the *k*_2_ value generated for ibuprofen. Uptake curves displayed a poor fit (as shown for imipramine concentration in *G. pulex*, which was consistently under-estimated). In addition, uptake constants in Table 1 specifically showed significant lack-of-fit for all compounds except propranolol and formoterol (*p*-value > 0.05). In fitting the depuration data using the sequential approach, a zero to mildly increasing slope was observed overall for metoprolol due to a wider scatter of data. A *k*_2_ value could not therefore be calculated for metoprolol. The potential for model uncertainty highlighted in this study is significant from a regulatory perspective, especially for compounds such as imipramine that was determined to be accumulative using the sequential method and non-accumulative using the simultaneous method (European Commission, 2006).

### 3.3 Assessment of *k*_1_, *k*_2_ and *C*_w_ constancy

The OECD 305 model makes several assumptions that *C*_w_, *k*_1_ and *k*_2_ do not change over time. To assess the potential validity of the *k*_1_ constancy assumption in the first instance, *k*_1_ was derived at each time point accordingly (Crookes & Brooke, 2011). It should be noted that a potential limitation to this approach was that the equation to calculate *k*_1_ uses the *k*_2_ estimate from the depuration phase, but this was deemed sufficient to identify any trends in any variation observed. As the lack-of-fit tests of the simultaneous method showed significant lacks-of-fit a direct estimation of *k*_2_ from linear regression is used in Eq. (6) for simplicity and increased reliability. When plotted against time (Fig. 2), a clear reduction in *k*_1_ over the exposure period was observed (especially for imipramine and diclofenac). Some random variance was also observed, such as for propranolol, which resulted in a relatively constant average *k*_1_ value of 0.58 (± 0.23), as would be expected. The simultaneous and sequential models estimated its *k*_1_ value to be 0.54 and 0.62 L kg^− 1^ day^− 1^, respectively and therefore showed reasonable agreement. This observation is significant as propranolol showed no lack-of-fit in the uptake curve; therefore, the agreement indicates that a lack-of-fit arises from variable *k*_1_ values over time.

This suggests that a decreasing *k*_1_ trend is the cause of the poor model fits although it is possible that this may also be caused by a changing *k*_2_ value (giving an apparent decrease in *k*_1_) or variable exposure concentrations in the water. However, water was monitored during the course of the experiments to account for any losses (Fig. 2 & Table S1) and the only compound that showed any significant loss was imipramine (> 20% nominal concentration). The *k*_1_ and *k*_2_ values should also be independent of pharmaceutical concentrations in the aqueous phase thus the trend observed is not in response to this variable (van Leeuwen & Vermeire, 2007). A change in *k*_2_ is likely to be represented as a decrease over time (unless the compound induces its own metabolism) assuming growth has a negligible effect and therefore would not account for decreases in *k*_1_. The elimination curves also showed no lack-of-fit for 6 compounds and the linear regression showed no trends of changing *k*_2_ values. The trend observed therefore is not in response to the parameters *C*_w_ or *k*_2_ and we therefore suggest the variability in uptake (decreasing *k*_1_) trend is the cause of the poor model fit.

### 3.4 Performance of OECD models using other micro-pollutant studies in *G. pulex*

*G. pulex* has been shown to metabolise organic compounds with low bioaccumulation factors previously observed (< 1500) (Ashauer et al., 2012). As defined by Annex XIII of the REACH criteria, for a compound to be considered bioaccumulative the BCF/BAF should be > 2000 (European Commission, 2006). Other work by Ashauer et al. investigated the toxicokinetics of 14 micro-pollutants in *G. pulex* and presented higher BCFs for three polychlorophenols in particular (Ashauer et al., 2010). As discussed by the authors, correlations showed an observable lack-of-fit in some cases. A slightly different model to the OECD 305 model was used in this work, where changes in *C*_w_ were accounted for as well as inclusion of an extra statistical algorithm to select the best parameter combinations of *k*_1_ and *k*_2_. However, when applying the OECD 305 models to BAF prediction, it is important to understand whether this is likely to be inaccurate and, amongst other reasons, potentially due to variation in *k*_1_, *k*_2_ or *C*_w_. To determine if any similar trends could be identified in other published *G. pulex* toxicokinetics studies, raw data from Ashauer et al., was re-examined using the OECD 305 modelling approach and presented in Table 2 (Ashauer et al., 2010). Although the authors' experiments were originally designed for determination of bioaccumulation, the report showed this to account for a small percentage of accumulation. Therefore, feeding was not included in any calculations. Similar to our findings for pharmaceuticals, both models displayed a statistically significant lack-of-fit for these organic micro-pollutant compounds. When the sequential method was applied, better fits were obtained for the depuration phase in comparison to the uptake phase. However, despite models used herein not performing as well overall, there was good agreement between the predicted BCF values and those generated by Ashauer et al. The data was then used to plot *k*_1_ versus time (Fig. S2), and again an obvious systematic decrease was observed for 9 out of 14 compounds. Statistical lack-of-fits (*p* < 0.05) were observed in the sequential uptake model especially for 4-nitrobenzylchloride, ethylacrylate, diazinon, aldicarb and hexachlorobenzene (*p* < 0.001). The latter two compounds were notable cases where the spread of replicate *k*_1_ data at each time-point was especially narrow and so the trend in *k*_1_ reduction over time was apparent. Of the remaining five compounds, trends in *k*_1_ were less evident and were coupled with *p* > 0.05 for lack-of-fit for four compounds using the sequential uptake model. The remaining compound, 2,4-dichlorophenol, showed no obvious trends in *k*_1_ variance as the major reason for the observed lack-of-fit in the uptake phase. In summary, *k*_1_ data could be considered reliable for only 5 of 14 compounds using the OECD 305 sequential model. In addition to the data of these 14 different organic pollutants, we also reassessed data from an exposure study of chlorpyrifos across 15 different invertebrate species to assess the issue more broadly (Rubach et al., 2010). Decreases in *k*_1_ were observed in several species and the trend was somewhat similar, albeit with larger scatter of the data (Fig. S3). This also identifies a further limitation that metabolism is likely to affect *k*_1_ and *k*_2_ values thus the differences in *k*_1_ constancy between organisms may be as a result of biotransformation. The study showed considerable differences between species in uptake and elimination rates showing that species type may affect the constancy of *k*_1_ in particular and further studies are required using more compounds between different species to fully assess this possibility. If the assumption of *k*_1_ constancy varies on a compound-by-compound basis, curve fitting methods to predict BCF are likely to be inherently inaccurate for environmental risk assessment purposes for *G. pulex*. Therefore, it is suggested that the approach taken herein (Crookes & Brooke, 2011) could be used to check the reliability of BCF data where a statistical lack-of-fit exists for this species.

Decreasing *k*_1_ could be explained by several possible mechanisms. The first is that growth dilution could cause an apparent decrease in *k*_1_ due to the mass of the organism increasing while the concentration of substance remains the same. However, this situation is unlikely given the short timescales of this work and that of Ashauer et al. (Ashauer et al., 2010). Therefore, growth of *G. pulex* is assumed to be negligible, particularly as this is regulated in line with their moulting cycle. However, further investigation would be required to fully support this. A second possibility is that *G. pulex* have been shown to alter respiration rates in the presence of a poor diet (Graça et al., 1993). As the animals were not fed during these experiments, it is possible that this slowed uptake. However, the toxicokinetic experiments by Ashauer et al. involved feeding organisms over their uptake period suggesting the uptake trend is not in response to diet induced factors (Ashauer et al., 2010). As these compounds are exposed to non-target animals, it is also possible that toxicodynamic effects could affect uptake, which is more easily interpreted using the dataset by Ashauer et al., where the exposure concentration was between 2 and 88 fold below the 24 h LC_50_ value. However for our dataset, mortality was not significantly higher than in controls for pharmaceuticals at the exposure concentrations used. Another consideration is that instantaneous sorption to the animal cuticle could account for the initially high *k*_1_ constants. However, an examination of the decrease in uptake rate against log*D* and log*P* revealed no correlation and compounds displayed independent *k*_1_ decreases (Fig. S4). However, log*D* only governs sorption to a certain extent and other physicochemical properties including polar/topological surface area, ionic state, amongst others, could influence sorption onto the exoskeleton. Where animals shed their exoskeleton during the exposure period, these were collected, weighed wet and radioactivity measured in a brief experiment. It was found that the maximum concentration of five of the eight pharmaceuticals on the exoskeleton material recovered did not exceed 24% of total compound mass in the animal in these cases (Table S3). Therefore, reduction in *k*_1_ via this mechanism is indeed plausible, but extended measurements across more compounds, conditions and replicates are recommended for full characterisation of this process. The potential for sorption as the reason for changes in *k*_1_ is not based on physiology, but rather on the physico-chemical properties of the xenobiotic itself, suggesting that rate constant stability may be compound specific.

## 4 Conclusions

This work demonstrates the importance of data interpretation using multiple modelling methods to estimate BCFs. Specifically, the comparative assessment of model lack-of-fits for both simultaneous and sequential models (where *k*_2_ remains constant) is recommended to reliably estimate and to ensure the accuracy of xenobiotic risk assessments. A decreasing trend in the uptake rate constant over time was apparent which disrupted the validity of the standard model assumptions tested, and suggests that more complex models are needed to describe accumulation of xenobiotics in invertebrates, more particularly in *G. pulex*. Kinetic BCF/BAF are an estimate of steady-state values, but it is possible that these models are adequate enough to indicate whether a compound may have a potential to accumulate or not. It is now important to identify whether such trends are also observed more generally across different species as well as a fuller investigation into the roles sorption and metabolism have in these standard models.

## Figures and Tables

**Table 1:** Toxicokinetic parameters and bioconcentration factors for eight PPCPs.

Compound	Simultaneous BCF						Sequential BCF^a^							Sequential BCF^b^					
*k*_1_ (L kg^− 1^ day^− 1^)	SE	*k*_2_ (day^− 1^)	SE	*p*-Value	BCF	*k*_1_ (L kg^− 1^ day^− 1^)	SE	*p*-Value	*k*_2_ (day^− 1^)	SE	*p*-Value	BCF	*k*_1_ (L kg^− 1^ day^− 1^)	SE	*p*-Value	*k*_2_ (day^− 1^)	*r*^2^	BCF
Propranolol	0.538	0.068	0.017	0.004	0.266	32	0.618	0.047	0.831	0.016	0.006	0.132	39	0.604	0.045	0.860	0.015	0.490	42
Formoterol	0.408	0.093	0.029	0.009	0.335	14	0.451	0.051	0.914	0.025	0.014	0.279	18	0.357	0.040	0.942	0.011	0.121	33
Imipramine	1.408	0.205	0.007	0.004	0.008	212	1.361	0.177	0.017	0.000	0.004	0.490	3811	1.360	0.177	0.017	0.000	0.001	4533
Metoprolol	0.076	0.022	0.005	0.008	0.073	16	N/A												
Terbutaline	0.136	0.020	0.011	0.004	0.026	12	0.135	0.016	0.027	0.006	0.003	0.381	22	0.117	0.016	0.027	0.006	0.200	19
Ranitidine	0.479	0.126	0.028	0.011	0.005	17	0.310	0.071	0.007	0.004	0.006	0.192	81	0.301	0.070	0.007	0.003	0.015	112
Diclofenac	0.273	0.037	0.020	0.005	0.002	14	0.253	0.025	0.017	0.009	0.003	0.156	27	0.269	0.026	0.023	0.013	0.243	21
Ibuprofen	0.338	0.094	0.012	0.008	0.000	27	0.582	0.097	0.013	0.022	0.014	0.004	27	0.488	0.073	0.029	0.010	0.093	50

*p*-Values were assessed via standard error (SE) and lack-of-fit tests.

a Sequential using curve fitting method.

b Sequential using linear regression.

**Table 2:** Toxicokinetic parameters and standard errors (SE) for 14 organic micropollutants with bioconcentrations factors and lack-of-fit tests for each compound.

Compound	BAF^a^	Simultaneous BCF						Sequential BCF						
*k*_1_ (L kg^− 1^ day^− 1^)	SE	*k*_2_ (day^− 1^)	SE	*p*-Value	BCF	*k*_1_ (L kg^− 1^ day^− 1^)	SE	*p*-Value	*k*_2_ (day^− 1^)	SE	*p*-Value	BCF
4-Nitrobenzyl chloride	185	666	665.740	4.540	4.540	0.000	147	259	28.324	0.00	1.212	0.149	0.230	214
2,4-Dichloroaniline	56	140	20.073	2.830	0.465	0.000	50	70	4.689	0.03	0.392	0.092	0.868	179
2,4-Dichlorophenol	4466	600	34.196	0.066	0.024	0.000	9050	750	48.646	0.00	0.010	0.018	0.400	72,728
4,6-Dinitro-o-cresol	37	39	2.610	1.146	0.124	0.070	34	37	1.595	1.00	0.729	0.077	0.033	51
1,2,3-Trichlorobenzene	191	1142	403.773	10.648	3.781	0.513	107	167	32.257	0.06	0.475	0.300	0.986	351
2,4,5-Trichlorophenol	2635	941	79.280	0.252	0.064	0.001	3729	1091	109.906	0.21	0.131	0.039	0.001	8327
Aldicarb	2	16	1.421	10.419	0.938	0.000	2	3	0.245	0.00	0.936	0.140	0.003	3
Carbofuran	65	10	0.355	0.146	0.019	0.026	68	10	0.570	0.11	0.140	0.019	0.025	72
Diazinon	82	276	24.271	3.569	0.3264	0.005	77	161	10.418	0.00	1.590	0.287	0.259	101
Ethylacrylate	87	110	12.139	1.594	0.2446	0.000	69	67	5.122	0.00	0.204	0.033	0.000	331
Hexachlorobenzene	2915	553	36.8714	0.221	0.046	0.000	2505	631	56.518	0.00	0.152	0.031	0.637	4160
Imidacloprid	7	2	0.093	0.265	0.038	0.000	7	2	0.117	0.02	0.175	0.022	0.000	13
Malathion	114	86	6.782	0.721	0.113	0.000	120	80	5.059	0.02	0.378	0.072	0.001	212
Sea nine	1732	755	57.821	0.303	0.065	0.000	2491	950	69.141	0.05	0.123	0.020	0.084	7696

a Reported from Ashauer et al. (2010).
