# Supplementary material for: Assessing the reliability of uptake and elimination kinetics modelling approaches for estimating bioconcentration factors in the freshwater invertebrate, Gammarus pulex
Source: Sci Total Environ. 2016 Mar 15;547:396–404. doi: 10.1016/j.scitotenv.2015.12.145 (PMC4956724; doi:10.1016/j.scitotenv.2015.12.145)
Supplement: Supplementary file 1 — Supplementary material. [file mmc1.docx]

*Supplementary Information*

**ASSESSING THE RELIABILITY OF UPTAKE AND ELIMINATION KINETICS MODELLING APPROACHES FOR ESTIMATING BIOCONCENTRATION FACTORS IN THE FRESHWATER INVERTEBRATE, *GAMMARUS PULEX*.**

Thomas H. Miller^a^, Gillian L. McEneff^a^, Lucy C. Stott^b^, Stewart F. Owen^b^, Nicolas R. Bury^a^, Leon P. Barron^a^*

*^a^Analytical & Environmental Sciences Division, Faculty of Life Sciences and Medicine, King’s College London, 150 Stamford Street, London, SE1 9NH, United Kingdom.*

*^b^AstraZeneca, Global Environment, Alderley Park, Macclesfield, Cheshire SK10 4TF, UK*

*^c^Current Address: WCA, Brunel House, Volunteer Way, Faringdon, Oxfordshire, SN7 7YR*

*Corresponding author email: [leon.barron@kcl.ac.uk](mailto:leon.barron@kcl.ac.uk); Tel: +44 20 7848 3842; Fax: +44 20 7848 4980

Table of Contents

**S 1.0** Pharmaceutical concentrations in water S3

**S 2.0** Physico-chemical properties S4

**S 3.0** Estimates of *k*_2_ by linear regression S5

**S 4.0** Time plots of *k*_1_ for 14 organic micro-pollutants S6

**S 5.0** Time plots of *k*_1_ for 15 arthropod species S7

**S 6.0** Decrease of *k*_1_ against log*P* and log*D* S8

**S 7.0** Sorption of pharmaceuticals to *G. pulex* exoskeleton S9

*List of Tables*

**Table S1.** Water concentrations measured during the uptake and depuration phases of the toxicokinetic experiments

**Table S2.** Physico-chemical properties of pharmaceuticals used in exposures

**Table S3.** Sorption of pharmaceuticals to *G. pulex* exoskeleton

*List of Figures*

**Figure S1.** Linear regression curves to estimate *k*_2_

**Figure S2.** Measurement of *k*_1_ over time for 14 organic micro-pollutants

**Figure S3.** Measurement of *k*_1_ over time for 15 different arthropod species exposed to chlorpyrifos

**Figure S4.** Relative decrease of *k*_1_ against log*D* at exposure pH for pharmaceuticals and organic micropollutants

**S 1.0** Pharmaceutical concentrations in water

Table S1 presents the xenobiotic concentrations determined over the course of the uptake and depuration phases of the toxicokinetic experiments along with respective losses to the wells of the culture plates via sorption.

Table S1. Concentrations of xenobiotics in the uptake and depuration phases of the toxicokinetic experiments.

S 2.0 Physico-chemical properties

**Table S2.** Physico-chemical properties of the selected pharmaceuticals used in exposures

| Compound | ^a^Log*P* | ^a^Log*D*_8.1_ | Predominant Form |
| --- | --- | --- | --- |
| Propranolol | 3.3 | 1.8 | Cationic |
| Metoprolol | 1.9 | 0.4 | Cationic |
| Formoterol | 1.6 | 0.6 | Cationic |
| Terbutaline | 0.5 | -1.0 | Cationic |
| Imipramine | 4.7 | 3.3 | Cationic |
| Ranitidine | 1.9 | -1.0 | Cationic |
| Diclofenac | 4.5 | 1.0 | Anionic |
| Ibuprofen | 3.4 | 0.0 | Anionic |

^a^Calculated from ACD Labs Percepta Software

S 3.0 Estimates of *k*_2_ by linear regression

Figure S1. Linear regression curves to estimate *k*_2_

**S 4.0** Time dependency plots for *k*_1_ for 14 organic micro-pollutants

**Figure S2.** Measurement of *k*_1_ over time for 14 organic micro-pollutants (Ashauer et al., 2010)

**S 5.0** Time dependency plots for *k*_1_ across 15 arthropod species

***Anax imperator***

**Figure S3.** Measurement of *k*_1_ over time for 15 different arthropod species exposed to chlorpyrifos. Meta-analysis performed using data from Rubach et al., 2010.

**S 6.0** Decrease of *k*_1_ against log*P* and log*D*


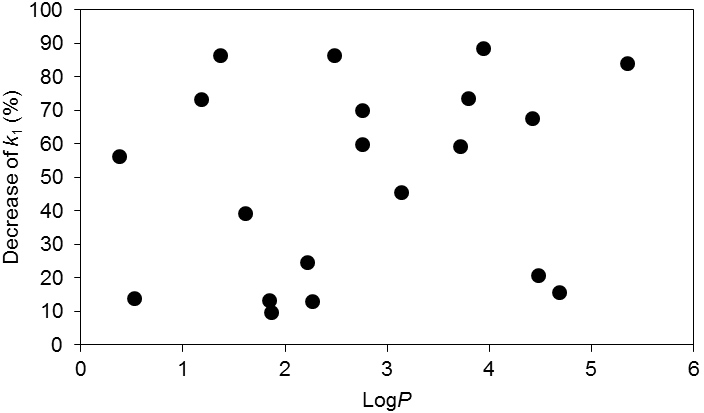

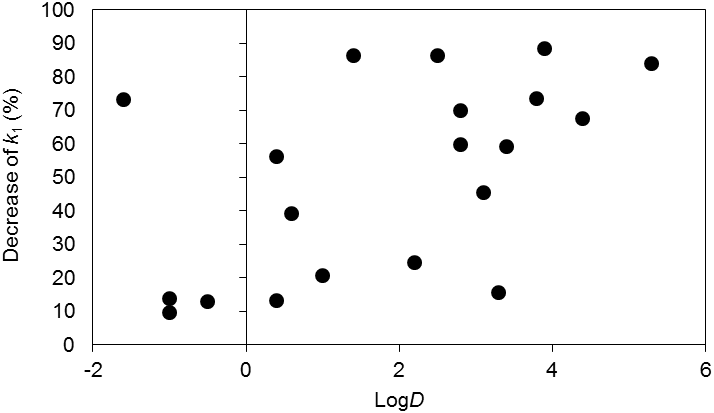


**Figure S4.** Relative decrease of *k*_1_ against log*P*/*D* at respective exposure pH for pharmaceuticals (this study) and organic micro-pollutants (Ashauer et al., 2010). Log*P*/*D* estimated by ACD Labs Percepta.

**S 7.0** Sorption of pharmaceuticals to *G. pulex* exoskeleton

**Table S3.** Sorption of pharmaceuticals to *G. pulex* exoskeleton

| Compound | Mean Compound  Mass per Animal | Total Mass Compound  on exoskeleton | Maximum concentration sorbed to exoskeleton |
| --- | --- | --- | --- |
|  | (n=3, pg) | (pg) | (%) |
| Propranolol | 336 | 44 (n=1) | 13 |
| Diclofenac | 2070 | 368 (n=4) | 18 |
| Imipramine | 1921 | 50 (n=2) | 3 |
| Ranitidine | 238 | 58 (n=1) | 24 |
| Metoprolol | 74 | 14 (n=2) | 19 |
|  |  |  |  |
